# Lysosomotropic agents including azithromycin, chloroquine and hydroxychloroquine activate the integrated stress response

**DOI:** 10.1038/s41419-020-03324-w

**Published:** 2021-01-06

**Authors:** Ai-Ling Tian, Qi Wu, Peng Liu, Liwei Zhao, Isabelle Martins, Oliver Kepp, Marion Leduc, Guido Kroemer

**Affiliations:** 1 Centre de Recherche des Cordeliers, Equipe labellisée par la Ligue contre le cancer, Université de Paris, Sorbonne Université, Inserm U1138, Institut Universitaire de France, Paris, France; 2 Metabolomics and Cell Biology Platforms, Gustave Roussy, Villejuif, France; 3 Department of Breast and Thyroid Surgery, Renmin Hospital of Wuhan University, Wuhan, Hubei P. R. China; 4 Suzhou Institute for Systems Medicine, Chinese Academy of Medical Sciences, Suzhou, China; 5 Pôle de Biologie, Hôpital Européen Georges Pompidou, AP-HP, Paris, France; 6 Karolinska Institutet, Department of Women’s and Children’s Health, Karolinska University Hospital, Stockholm, Sweden

**Keywords:** Macroautophagy, Viral infection

## Abstract

The integrated stress response manifests with the phosphorylation of eukaryotic initiation factor 2α (eIF2α) on serine residue 51 and plays a major role in the adaptation of cells to endoplasmic reticulum stress in the initiation of autophagy and in the ignition of immune responses. Here, we report that lysosomotropic agents, including azithromycin, chloroquine, and hydroxychloroquine, can trigger eIF2α phosphorylation in vitro (in cultured human cells) and, as validated for hydroxychloroquine, in vivo (in mice). Cells bearing a non-phosphorylatable eIF2α mutant (S51A) failed to accumulate autophagic puncta in response to azithromycin, chloroquine, and hydroxychloroquine. Conversely, two inhibitors of eIF2α dephosphorylation, nelfinavir and salubrinal, enhanced the induction of such autophagic puncta. Altogether, these results point to the unexpected capacity of azithromycin, chloroquine, and hydroxychloroquine to elicit the integrated stress response.

## Introduction

Azithromycin (AZT), chloroquine (CQ), and 3-hydroxychloroquine (HCQ) have attracted much attention over the past months as possible (and controversial) therapeutic agents for the treatment of coronavirus disease-19 (COVID-19)^1,2^. At this point, it has not been resolved whether the frequently administered combination regimen of AZT and HCQ (often supplemented with zinc) itself reduces the morbidity and mortality of COVID-19 or whether accompanying measures (such as provision of anti-diabetic, anti-hypertensive, anti-inflammatory, and/or anti-thrombotic agents) or even placebo effects account for the clinical efficiency of AZT + HCQ, which are more frequently observed in retrospective analyses and uncontrolled clinical studies^3–5^ than in prospective randomized studies^6–9^.

AZT is a macrolide antibiotic, while CQ and HCQ are antimalarial drugs. HCQ is also been widely used for the treatment of rheumatoid arthritis and systemic lupus erythematosus^10,11^. All the three agents are lysosomotropic^12–14^, meaning that they are sufficiently lipophilic to penetrate into cells but also weak bases so that they get protonated at low pH to become trapped in lysosomes, hence gradually increasing their concentration in the lysosomal lumen until they destabilize lysosomal membranes due to detergent-like effects, causing a loss of lysosomal acidification and blockade of lysosomal functions^15,16^ that ultimately activates homeostatic circuitries including the activation of transcription factors such as TFEB and TFE3 for lysosomal biogenesis^17^. In addition, the loss of lysosomal acidity/function observed in cells treated with AZT, CQ, or HCQ results in the blockade of lysosomal fusion with autophagosomes, thus stalling autophagic flux and causing the accumulation of autophagosomes that cannot be eliminated^18–20^. Moreover, CQ and HCQ can stimulate lysosomal membrane permeabilization that secondarily elicits the mitochondrial pathway of apoptosis^21^, hence resulting in cell death, likely contributing to the toxicity of these agents^22,23^.

The integrated stress response (ISR) consists in the phosphorylation of the phylogenetically conserved eukaryotic initiation factor 2α (eIF2α) by a series of eIF2α kinases (EIF2K1 to 4) and plays a cardinal role in the adaptation of stress to endoplasmic reticulum (ER) stress (in particular, the accumulation of unfolded or misfolded proteins in the ER lumen)^24^, in the innate cellular defense against viral infections (to block the translation of virus-encoded RNAs into protein)^25–27^, as well as in the initiation of autophagy (which also can lead to the elimination of intracellular pathogens)^28–31^. Moreover, eIF2α phosphorylation contributes to the phenomenon of “immunogenic cell death” (ICD)^32–34^, which likely plays a major role in connecting the virus-induced death of infected cells to immune response that ultimately lead to the active elimination of virus-infected cells by cytotoxic T lymphocytes^35–37^. This latter effect is achieved due to the contribution of eIF2α phosphorylation to (i) autophagy, which enables the lysosomal secretion of ATP (which is a major chemoattractant for dendritic cell precursors)^28,29,31,38^ and (ii) the exposure of the ER lumen protein calreticulin at the cell surface (where it acts as an eat-me signal to render dying/dead cells palatable to dendritic cells, allowing them to present viral antigens to T lymphocytes)^33,39–41^.

In view of the considerable (patho)physiological relevance of ISR, we decided to investigate whether AZT, CQ, or HCQ may induce this phenomenon. Here, we show that these three agents induce signs of ISR in vivo, and that ISR contributes to the accumulation of stalled autophagosomes as well as to the cytotoxicity of these agents.

## Results

### Lysosomotropic agents induce eIF2a phosphorylation in vitro

**Fig. 1:**
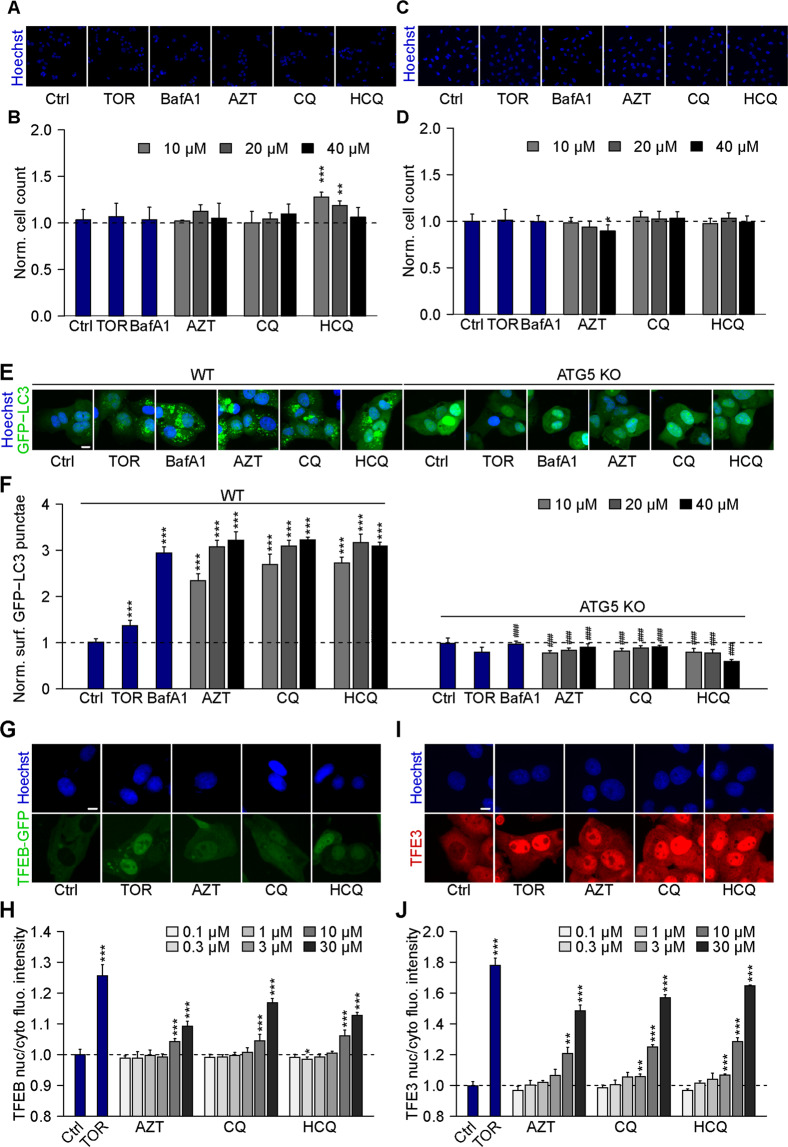
Chloroquine, hydroxychloroquine, and azithromycin induce the formation of LC3 puncta and trigger TFEB/TFE3 translocation. **A–D** Human osteosarcoma U2OS-GFP-LC3 (**A**, **B**) or human glioma H4-GFP-LC3 cells (**C**, **D**) were treated with chloroquine (CQ; 10, 20, 40 μM), hydroxychloroquine (HCQ; 10, 20, 40 μM), azithromycin (AZT; 10, 20, 40 μM), the autophagy inducer torin 1 (TOR; 300 nM), or the inhibitor of autophagic flux bafilomycin A1 (BafA1; 100 nM) for 6 h. After fixation, healthy cells depicted by normal nuclear morphology were enumerated. Representative microscopical images are shown in **A** and **C** (AZT, CQ, and HCQ, 40 µM) and normalized mean data are depicted as bar charts in **B** and **D**. Data are means ± SD of four replicates (*P < 0.05, **P < 0.01, ***P < 0.001 vs. vehicle control (Ctrl); Student’s *t*-test). **E**, **F** U2OS-GFP-LC3 wild type or ATG5 knockout (KO) cells were treated with CQ, HCQ, or AZT (all at 10, 20, 40 μM), TOR (300 nM), and BafA1 (100 nM) for 6 h. After fixation, GFP-LC3 dots were analyzed as a proxy for autophagy induction. Representative microscopical images are shown in **E** (AZT, CQ, and HCQ, 40 µM) and normalized mean data are depicted as bar chart in **F**. Data are means ± SD of four replicates (**P < 0.01, ***P < 0.001 vs. vehicle control (Ctrl), and ^###^*P* < 0.001 vs. WT; Tukey’s multiple comparisons test). **G**, **H** U2OS cells stably expressing GFP-TFEB fusion protein were treated with CQ, HCQ, or AZT (all at 0.1, 0.3, 1, 3, 10, 30 μM) for 6 h. TOR at 300 nM was used as a positive control for TFEB nuclear translocation. Images were analyzed and the ratio of GFP intensities in nuclei and cytoplasm was calculated to indicate TFEB translocation to nuclei (**H**). Representative images are depicted in **G** (AZT, CQ, and HCQ, 30 µM). **I**, **J** U2OS cells were treated as above, and then TFE3 translocation was assessed microscopically after immunostaining (**I**). TOR at 300 nM was used as a positive control for TFE3 nuclear translocation. TFE3 intensities in the nucleus and the cytoplasm were measured, and the nucleo-to-cytoplasmic ratio of TFE3 intensities was calculated to indicate nuclear translocation of TFE3 (**J**). Data are means ± SD of four replicates (*P < 0.05, **P < 0.01, ***P < 0.001 vs. Ctrl, Student’s *t*-test). Scale bars equal 10 μm.

**Fig. 2:**
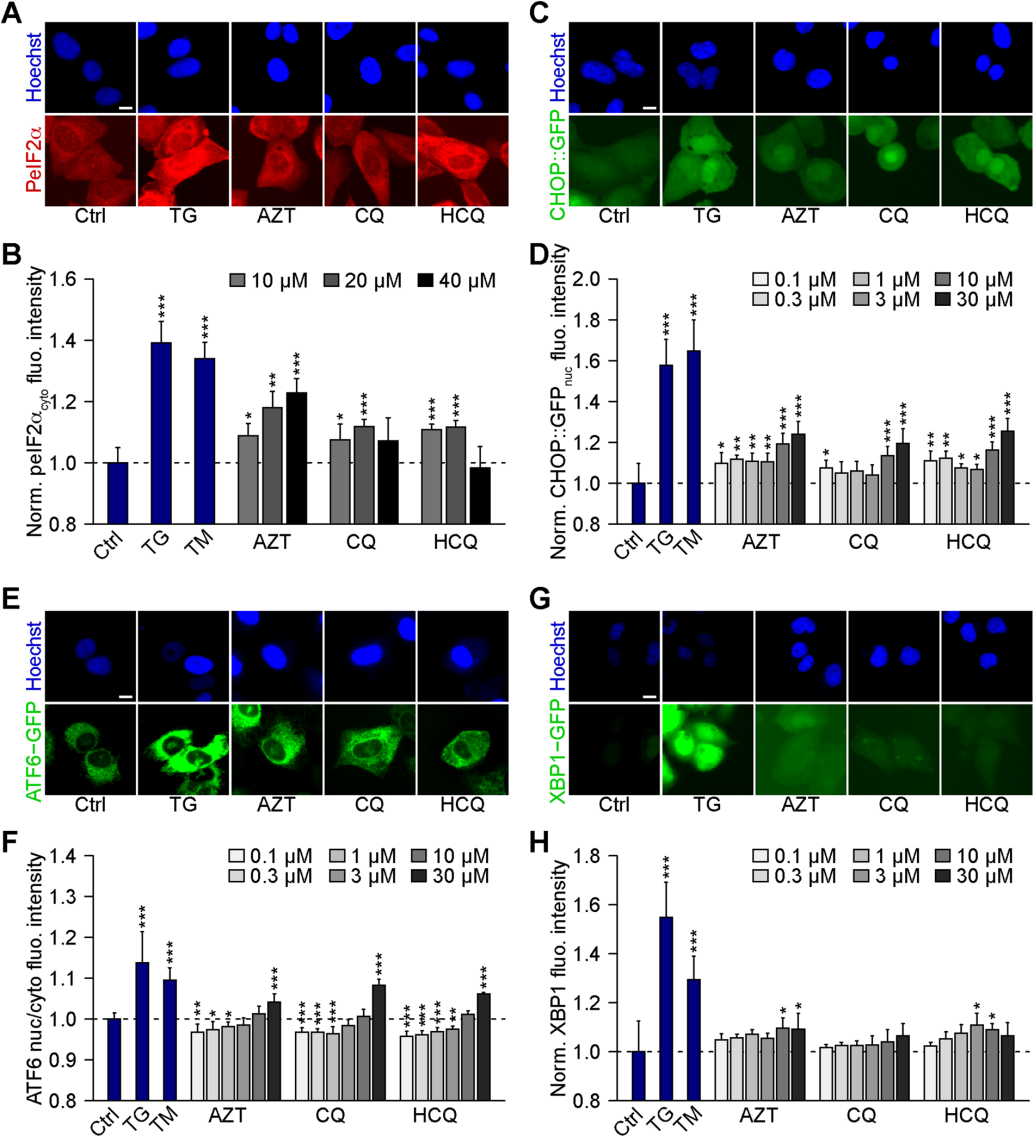
Chloroquine, hydroxychloroquine, and azithromycin induce ER stress. **A**, **B** Human osteosarcoma U2OS cells were treated with chloroquine (CQ), hydroxychloroquine (HCQ), and azithromycin (AZT; all at 10, 20, 40 μM) for 16 h, then fixed and imaged. Tunicamycin (TM, 3 μM) and thapsigargin (TG, 3 μM) were used as positive controls for ER stress induction. PeIF2α was assessed by means of an immunofluorescence staining using a phosphoneoepitope-specific antibody (**A**) and the cytoplasmic intensity is depicted (**B**) (AZT, CQ, and HCQ, 40 µM). **C**, **D** Human osteosarcoma U2OS cells stably expressing GFP under the promoter of DDIT3 (CHOP::GFP) were treated with the indicated agents (TM (3 μM), TG (3 μM), CQ, HCQ, or AZT (all at 0.1, 0.3, 1, 3, 10, 30 μM)) for 24 h. After fixation, CHOP::GFP fluorescence was assessed microscopically as shown in **C**, and the average nuclear intensity was quantified (**D**). E, **F** U2OS cells stably expressing GFP-ATF6 were treated with the indicated agents for 24 h. After the cells were fixed, GFP-ATF6 nuclear translocation was assessed as shown in **E** (AZT, CQ, and HCQ, 30 µM), and the nuclear-to-cytoplasmic ratio of GFP-ATF6 intensity was quantified (**F**). **G**, **H** U2OS cells stably expressing XBP1ΔDBD-venus (for monitoring venus expression upon alternative splicing of XBP1 mRNA) were treated as above for 24 h. After fixation, XBP1s expression was assessed via fluorescent microscopy as shown (**G**) (AZT, CQ, and HCQ, 30 µM), and the average intensity was measured (**H**). Data are means ± SD of four replicates (*P < 0.05, **P < 0.01, ***P < 0.001 vs. vehicle control (Ctrl), Student’s *t*-test). Scale bars equal 10 μm.

Human U2OS osteosarcoma cells stably expressing a GFP-LC3 fusion protein exhibit GFP-LC3 dots in the cytoplasm (corresponding to “autophagic puncta”)^42^ in response to the autophagy inducer torin1 (TOR, an inhibitor of mechanistic target of rapamycin, mTOR) and the lysosomal inhibitor bafilomycin A1 (BafA1, an inhibitor of the vacuolar-type H^+^-ATPase (V-ATPase) that is required for lysosomal acidification)^43^. Similar to BafA1, the three lysosomotropic agents AZT, CQ, and HCQ did not cause any cytotoxicity in the timeframe of the experiment (Fig. 1A–D) but stimulated a dose-dependent increase in GFP-LC3 dots. The formation of GFP-LC3 puncta was observed in wild-type U2OS and human glioma H4 cells but not in cells that are deficient for the essential autophagy protein ATG5 and which acts upstream of LC3 to facilitate lipidation and membrane association (Fig. 1E, F and Supplementary Fig. 1). Moreover, AZT, CQ, and HCQ stimulated the translocation of the transcription factors TFEB and TFE3 from the cytoplasm to the nuclei, as determined in U2OS cells expressing a GFP-TFEB fusion protein (Fig. 1G, H) or by immunofluorescence detection of TFE3 (Fig. 1I, J). AZT, CQ, and HCQ inhibited autophagic flux in U2OS RFP-GFP-LC3 tandem reporter cells, as can be expected from agents that perturb lysosomal function (Supplementary Fig. 2)^15,16,44^. In addition, AZT, CQ, and HCQ induced the phosphorylation of eIF2α (as measured by immunofluorescence and immunoblot using a phosphoneoepitope-specific antibody) (Fig. 2A, B and Supplementary Fig. 3)^45^, the activation of the transcription factor CHOP (as indicated by the expression of GFP placed under the control of the CHOP promoter) (Fig. 2C, D), the upregulation of ATF6 (as indicated by the expression of an ATF6-GFP fusion protein) (Fig. 2E, F), and the activation of XBP1 (as indicated by the expression of an XBP1-GFP/Venus fusion protein in which GFP/Venus is only expressed after that IRE1α has caused the splicing of the corresponding mRNA (Fig. 2G, H). However, in quantitative terms, the effects of AZT, CQ, and HCQ on CHOP, ATF6, and XBP1 appear relatively minor when compared to the positive controls thapsigargin and tunicamycin employed to elicit ER stress (Fig. 2C–H). Only the level of eIF2α phosphorylation induced by AZT, CQ, and HCQ reaches that of the positive controls (Fig. 2A, B). Similarly, CQ and HCQ (but not AZT) induced a relatively low level of NF-kB activation as compared to the positive control, tumor necrosis factor-α (Supplementary Fig. 4). We conclude that AZT, CQ, and HCQ are potent perturbators of lysosomal function as well as potent inducers of the ISR consisting in eIF2α phosphorylation.

### eIF2a phosphorylation is required for the induction of autophagic puncta

**Fig. 3:**
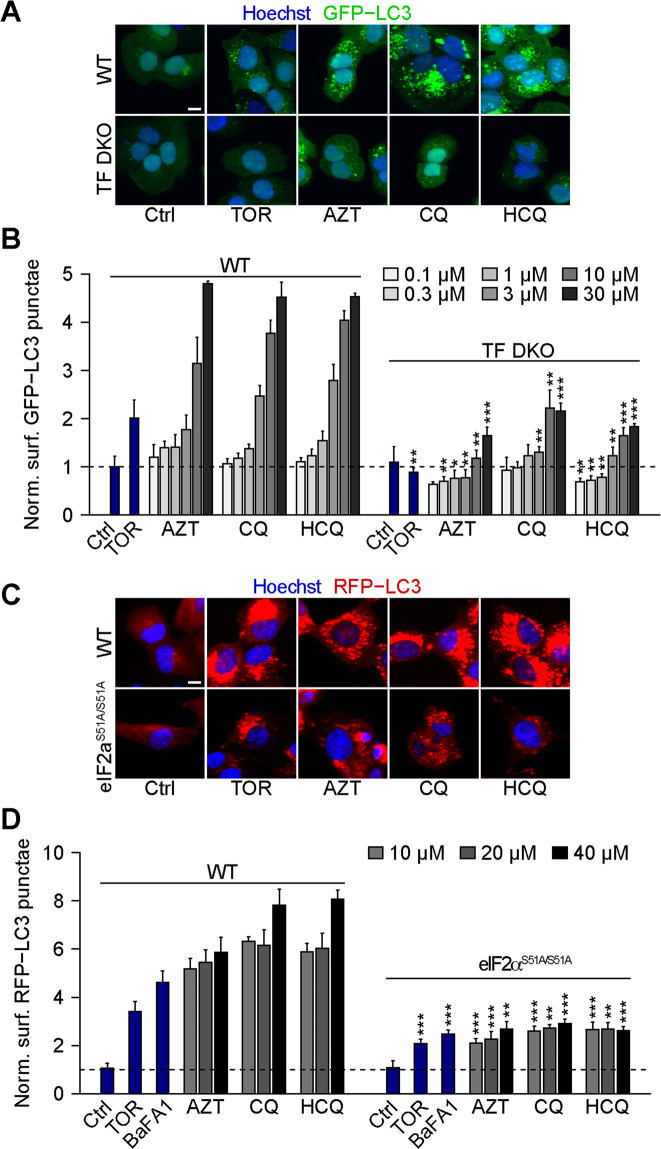
Chloroquine, hydroxychloroquine, and azithromycin-induced autophagy depends on TFEB/TFE3 and eIF2α. **A**, **B** Human osteosarcoma U2OS wild type (WT) or TFEB/TFE3 double KO (TF DKO) cells both stably expressing GFP-LC3 were treated with the indicated compounds (torin 1 (TOR; 300 nM), chloroquine (CQ), hydroxychloroquine (HCQ), and azithromycin (AZT; all at 0.1, 0.3, 1, 3, 10, 30 μM)) for 6 h. After fixation, GFP-LC3 dots were analyzed as a proxy for autophagy. Representative images are depicted in **A** (AZT, CQ, and HCQ, 30 µM) and normalized data are shown as bar chart in **B**. Data are means ± SD of four replicates (**P < 0.01, ***P < 0.001 vs. WT; Student’s *t*-test). **C**, **D** U2OS WT or PeIF2α S51A knockin cells both expressing RFP-LC3 were treated as indicated with TOR (300 nM), bafilomycin A1 (BafA1, 100 nM), CQ, HCQ, and AZT (all at 10, 20, 40 μM) for 6 h. After fixation, RFP-LC3 dots were analyzed by fluorescent microscopy. Representative images are shown in **C** (AZT, CQ, and HCQ, 40 µM) and normalized data are quantitated as a bar plot in **D**. Data are means ± SD of four replicates (**P < 0.01, ***P < 0.001 vs. WT; Student’s *t*-test). Scale bars equal 10 μm.

**Fig. 4:**
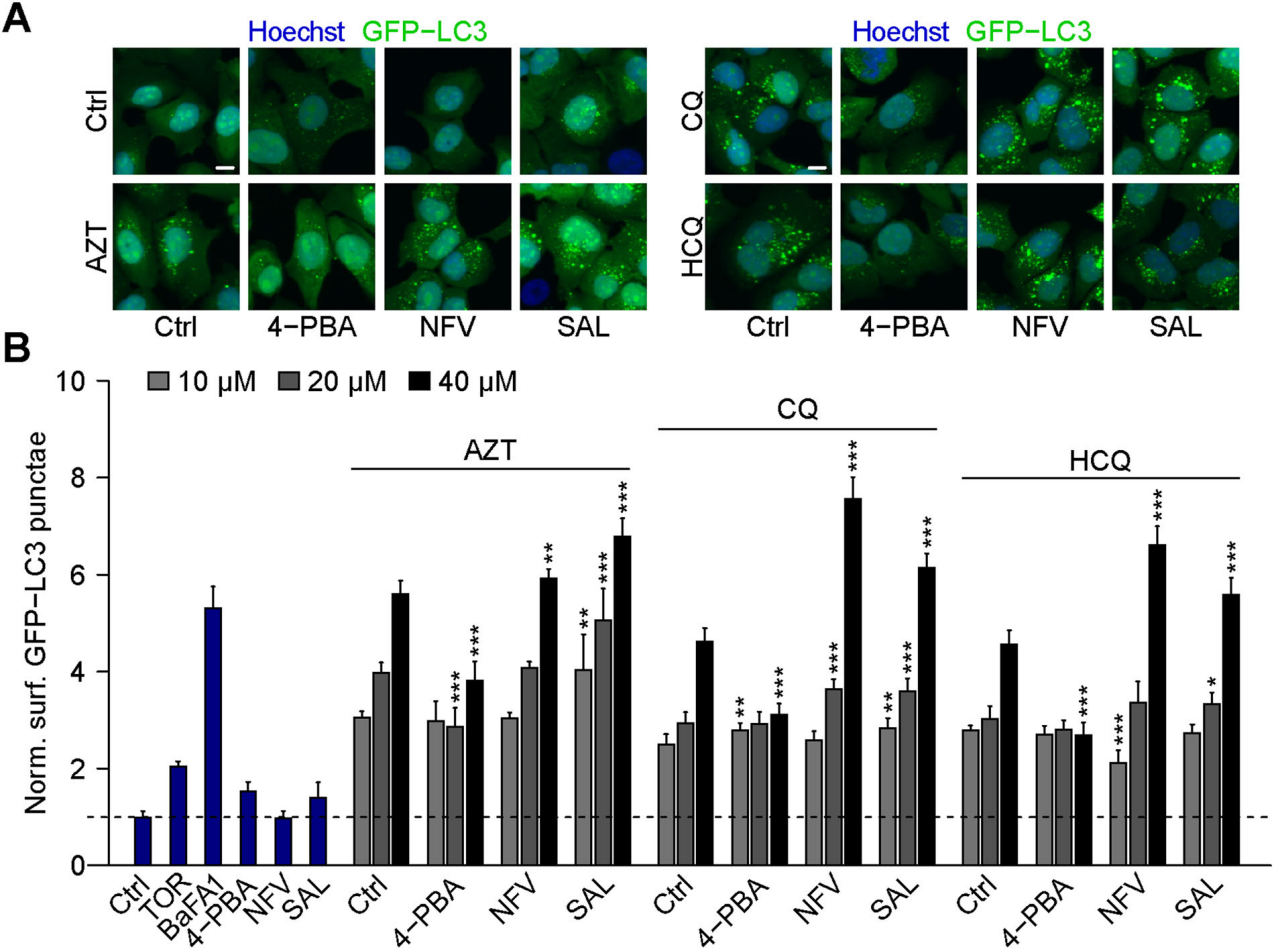
eIF2α phosphatase inhibitors increase autophagy induced by chloroquine, hydroxychloroquine, and azithromycin. **A**, **B** Human osteosarcoma U2OS cells stably expressing GFP-LC3 were treated with 5 mM 4-phenylbutyric acid (4-PBA), 10 µM nelfinavir (NFV), 25 µM salubrinal (SAL), or were left untreated for 6 h in the presence or absence of 10, 20, 40 μM chloroquine (CQ), hydroxychloroquine (HCQ), or azithromycin (AZT). After fixation, GFP-LC3 dot formation was analyzed by microscopy. Torin (TOR) at 300 nM was used as a positive control for autophagy induction and bafilomycin A1 (BafA1) at 100 nM was used as an inhibitor of autophagic flux. Representative images are depicted in **A** (AZT, CQ, and HCQ (30 µM) alone or in combination with eIF2α phosphatase inhibitors) and normalized data are shown as a bar charts in **B**. Data are means ± SD of four replicates (*P < 0.05, **P < 0.01 ***P < 0.001 vs. solvent control (Ctrl), Student’s *t*-test). Scale bars equal 10 μm.

TFEB and TFE3 are well known pro-autophagic transcription factors^46,47^. Accordingly, their double knockout (DKO) attenuated the induction of GFP-LC3 puncta by AZT, CQ, and HCQ (Fig. 3A, B). Many autophagy inducers require eIF2α phosphorylation as a mandatory step for the ignition of the process^48^. Accordingly, we observed that a knockin mutation that renders eIF2α non-phosphorylatable (due to the replacement of serine in position 51 by an alanine residue: genotype *eIF2*α^S51A/S51A^) strongly inhibited the induction of GFP-LC3 puncta by AZT, CQ, and HCQ (Fig. 3C, D). Similarly, inhibition of ER stress with the chemical chaperone 4-phenylbutyric acid (4-PBA)^49^ attenuated the induction of GFP-LC3 puncta by AZT, CQ, and HCQ (Fig. 4A, B). Conversely, treatment of the cells with two inhibitors of eIF2α dephosphorylation, nelfinavir^50^ and salubrinal^51^, enhanced the formation of GFP-LC3 puncta in response to AZT, CQ, and HCQ (Fig. 4A, B and Supplementary Fig. 5).

**Fig. 5:**
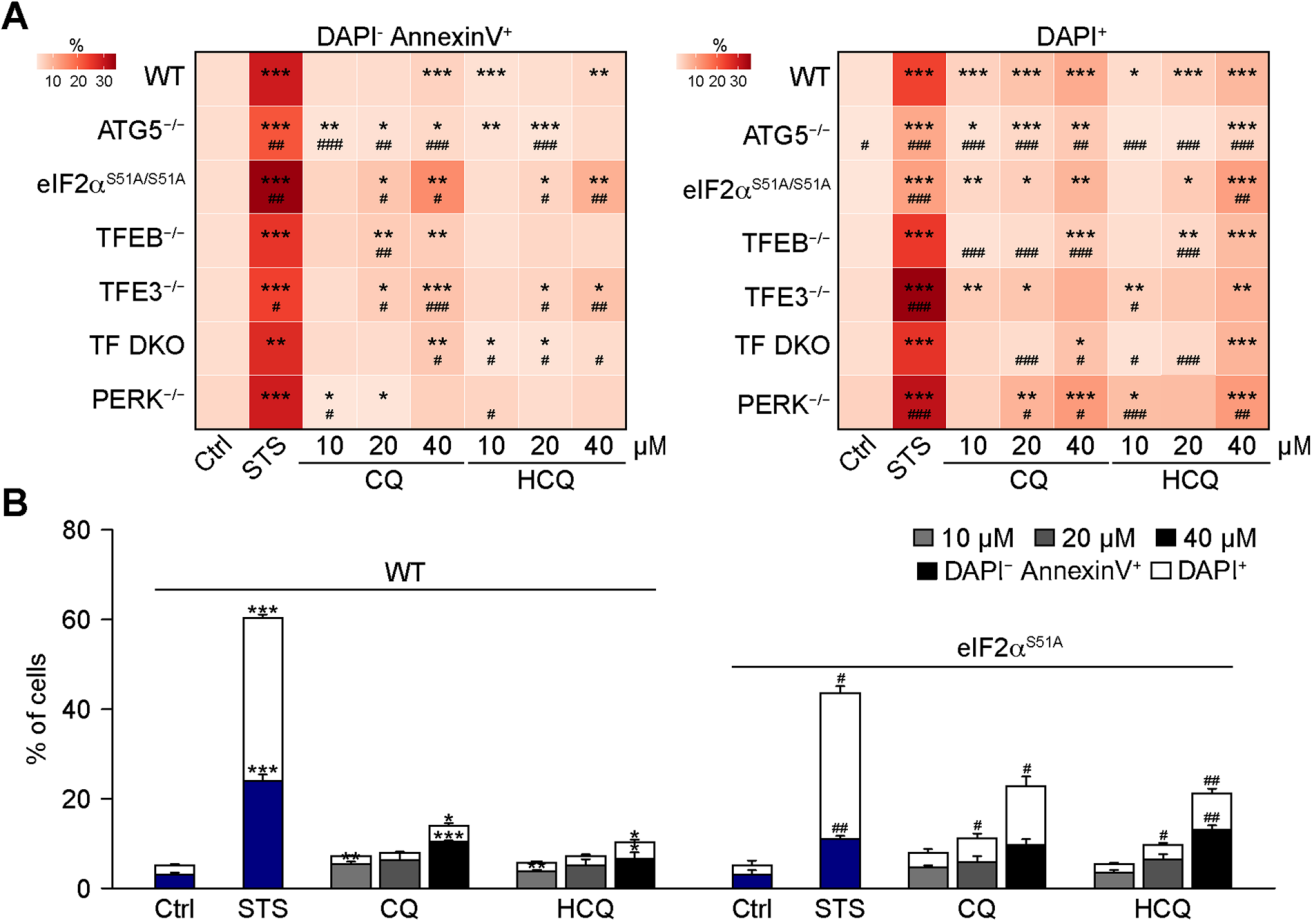
Increase in toxic effect of chloroquine and hydroxychloroquine in eif2α mutant cells. Human osteosarcoma U2OS either WT, ATG5^−^^/−^, TFEB^−^^/−^, TFE3^−/−^, TF DKO, PERK^−/−^ or carrying an eIF2α^S51A/S51A^ knockin mutation were treated with 10, 20, or 40 μM of chloroquine (CQ) or hydroxychloroquine (HCQ) for 24 h. Plasma membrane integrity loss and phosphatidylserine (PS) exposure (with) were measured by flow cytometry employing DAPI and AlexaFluor 647-coupled annexin V, respectively. DAPI^+^ and Annexin V^+^ DAPI^−^ cellular populations were quantified and are depicted as a heatmap **A**. Data are means ± SD of three replicates (*P < 0.05, **P < 0.01, ***P < 0.001, ^#^*P* < 0.05, ^##^*P* < 0.01, ^###^*P* < 0.001 vs. WT, Student’s *t*-test). Data for WT and eIF2α^S51A^ expressing mutant U2OS are depicted as bar chart in **B**. Staurosporine (STS) at 2 μM was used as a positive control for cell death induction. Data are means ± SD of three replicates (*P < 0.05, **P < 0.01, ***P < 0.001, ^#^*P* < 0.05, ^##^*P* < 0.01, vs. vehicle control (Ctrl), Student’s *t*-test).

In accord with previous work^21^, CQ and HCQ induces some degree of cellular toxicity, leading to the manifestation of apoptotic and necrotic events that can be distinguished by dual staining with annexin-V-FITC (which stains apoptotic and necrotic cells) the vital dye 4′,6-diamidino-2-phenylindole (DAPI, which only stains necrotic cells)^52^. Among the genotypes evaluated in this paper (*ATG5*^−/−^, *eIF2*α^S51A/S51A^, *TFEB*^−/−^, *TFE3*^−/−^, *TFEB*/*TFE3* DKO, *PERK*^−/−^) the *eIF2*α^S51A^ knockin mutation rendering eIF2α non-phosphorylatable had the strongest effect on apoptosis induction by CQ and HCQ (Fig. 5A), increasing cellular killing by CQ and HCQ but not by the general tyrosine kinase inhibitor and apoptosis inducer staurosporin (STS) (Fig. 5B and Supplementary Fig. 6). These results point to the ISR as central for the effects of CQ and HCQ.

### Lysosomotropic agents induce eIF2α phosphorylation in vivo

**Fig. 6:**
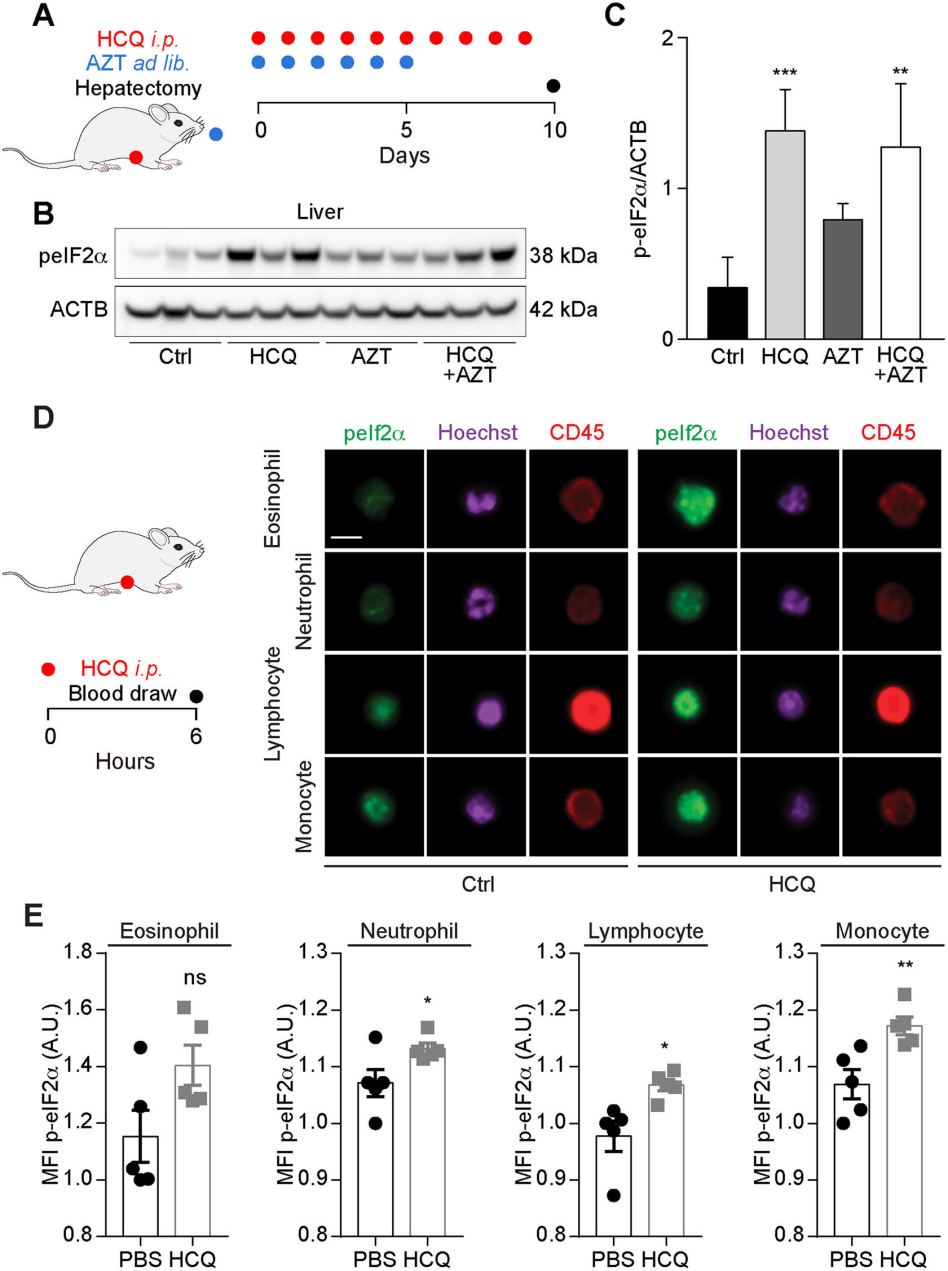
Effects of hydroxychloroquine plus azithromycin in vivo. Mice were treated intraperitoneally (i.p.) with 50 mg/kg/day hydroxychloroquine (HCQ), orally fed with azithromycin (AZT) (3 mg/L in drinking water), or their combination as illustrated in the scheme (**A**). Livers were excised from three mice by group at the end of the treatment and the tissues were subjected to protein extraction for SDS–PAGE and immunoblot to detect the phosphorylation of peIF2α (**B**). β-Actin (ACTB) was used as a loading control. Band intensities were quantified by densitometry and the ratio of peIF2α to ACTB was calculated. Data are expressed as means ± SEM of three mice (**C**). Statistical significance is indicated as **P < 0.01 and ***P < 0.001 as compared with untreated control (Ctrl) (Student’s *t*-test). **D, E** Mice were treated with 50 mg/kg HCQ i.p. and blood was collected after 6 h to determine the level of peIF2α by immunofluorescence and image flow cytometry in the depicted leukocyte populations. Representative images are shown in **D**. The scale bar equals 10 μm. Data are expressed as mean fluorescens intensities (MFI) means ± SEM of five mice (**E**). Statistical comparisons were done by two-tailed unpaired Student’s *t*-test (**E**) comparing HCQ-treated to control mice that received PBS (*P < 0.05, **P < 0.01).

The aforementioned results have been obtained in vitro, calling for their in vivo validation. For this, we injected mice intraperitoneally with HCQ (at a dose that inhibits autophagic flux)^53–56^ alone or in combination with AZT (supplemented in the drinking water). Of note, HCQ (but less so AZT) induced a remarkable and significant increase in eIF2α phosphorylation that was detectable by immunoblot in liver extracts (Fig. 6A, B) but less so in the myocardium (Supplementary Fig 7). In addition, one single injection of HCQ was able to stimulate a significant increase in eIF2α phosphorylation in several circulating leukocyte subsets (in particular neutrophil granulocytes, lymphocytes, and monocytes), as determined by immunofluorescence staining and imaging flow cytometry (Fig. 6D, E). Thus, HCQ can induce eIF2α phosphorylation in vivo, supporting the capacity of this agent to activate ISR.

## Discussion

As we show in this work, lysosomotropic agents including AZR, CQ, and HCQ are capable of stimulating the ISR. The capacity of these agents to induce the cardinal hallmark of ISR, eIF2α phosphorylation, is observed at similar concentrations as those required to induce the accumulation of autophagic puncta and to activate the transcription factors TFEB and TFE3 in a dose of 10–40 µM. The accumulation of autophagic puncta induced by AZT, CQ, and HCQ requires the initial steps of autophagy, as illustrated by the fact that ATG5-deficient cells fail to demonstrate this phenomenon. This is in accordance with findings showing that CQ can induce non-canonical V-ATPase-dependent LC3 lipidation^57^. Moreover, AZT, CQ, and HCQ were unable to elicit the accumulation of LC3-binding autophagosomes in cells expressing a non-phosphorylatable mutant of eIF2α, suggesting causality between ISR and the observed phenomenon. This conjecture was further supported by the observation that two inhibitors of the dephosphorylation of eIF2α enhanced autophagosome accumulation in vitro. Moreover, the apoptosis-inducing effect of CQ and HCQ was reduced in cells bearing mutant eIF2α.

The ISR plays a major role in the inhibition of viral replication. Indeed, multiple viruses have developed strategies to subvert the ISR, either by directly inhibiting eIF2α kinases or by deploying a decoy that resembles eIF2α, hence preventing the phosphorylation of the cellular protein^25,58,59^. In addition, a protein encoded by coronavirus counteracts the ISR at its very core by acting as a competitive inhibitor of the phospho-eIF2α-eIF2β interaction^27^. Hence, the question comes up whether the reported in vitro antiviral effects of CQ and HCQ^27,60^ are linked to their capacity to elicit the ISR, thus augmenting an innate immune response (such as the initiation of a type-1 interferon response^61,62^, beyond their action on acidophilic cellular compartments^63–65^.

The ISR also has a fundamental role in ICD. In a plausible scenario, cells infected by viruses ultimately succumb to viral infection. If the virus (or other agents) induce the ISR, cell death would be perceived as immunogenic, hence favoring the stimulation of an immune response that involves dendritic cells as antigen presenters that then “educate” cytotoxic T lymphocytes to recognize MHC class I-restricted viral peptides expressed on the surface of infected cells^37,66^. By clearing infected cells, the immune system then can remove all virus-replicative niches from the body to subsequently establish a memory response that protects the patient from challenges by the same or antigenically similar viruses.

We have found in the past that artificial induction of the ISR by agents that stimulate an ER stress response (such as thapsigargin injected into tumors) or inhibit the dephosphorylation of eIF2α (such as salubrinal and a peptides inhibiting the phosphatase PP1 interacting with its cofactor GADD34) can vigorously stimulate anticancer immune responses linked to ICD^67–70^. In this context, it is noteworthy that agents that selectively stimulate ISR but not any other manifestation of the unfolded stress response (such as the activation of ATF6 and that of IRE1/XBP1) are more efficient ICD inducers than agents with a broad effect on several arms of the unfolded stress response^33,71,72^. In quantitative terms, when compared to appropriate positive controls (thapsigargin, tunicamycin), AZT, CQ, and HCQ induced a strong ISR but scarce ATF6 and IRE1/XBP1 activation. Hence, these lysosomotropic agents induce a pattern of response that would be compatible with a pro-ICD action. However, further virological and immunological experimentation will be required to (in)validate this conjecture.

In essence, our results demonstrate that AZT, CQ, and HCQ stimulate the ISR. This might contribute to the potential antiviral and immunostimulatory effects of such lysosomotropic agents. However, to definitively prove the mechanistic relevance of such effects, it would be necessary to develop small animal models^73^ in which AZT, CQ, and HCQ, alone or in combination would have significant and reproducible antiviral activity.

## Materials and methods

### Cell culture and chemicals

Culture media and supplements for cell culture were purchased from Gibco-Life Technologies (Carlsbad, CA, USA) and plastic ware came from Greiner Bio-One (Kremsmünster, Austria) and Corning (Corning, NY, USA). Wild-type human osteosarcoma U2OS or human glioma H4 cells were purchased from the American Type Culture Collection (ATCC, Rockefeller, MD, USA), their derivatives stably expressing GFP-LC3, RFP-LC3, or RFP-GFP-LC3 were cultured in Dulbecco’s modified Eagle’s medium (DMEM) supplemented with 10% (v/v) fetal bovine serum (FBS), 10 U mL^−1^ penicillin sodium, and 10 μg mL^−1^ streptomycin sulfate at 37 °C in a humidified atmosphere with 5% CO_2_. TFEB-deficient (TFEB^−/−^), TFE3-deficient (TFE3^−/−^), TFEB and TFE3-double deficient (TF DKO), ATG5-deficient (ATG5^−/−^), and PERK-deficient (PERK^−/−^) U2OS cells were generated by means of CRISPR/Cas9-mediated genome editing, as per the manufacturer’s recommendations^31,74^. U2OS cells stably expressing RFP-LC3 bearing a non-phosphorylatable mutant of eIF2α (eIF2α^S51A^) were constructed by means of CRISPR/Cas9 knockin as previously detailed^31^. In addition, U2OS cells stably expressing GFP-TFEB, a GFP under the DDIT3 promoter (CHOP::GFP), GFP-ATF6, and XBP1s-ΔDBD-venus were generated by our group in the past^33,74^. Chloroquine diphosphate salt (CQ, #C6628), hydroxychloroquine sulfate (HCQ, #PHR1782), azithromycin (AZT, #75199), 4-phenylbutyric acid (4-PBA, #P21005), nelfinavir (NFV, #CDS021783), salubrinal (SAL, #324895), thapsigargin (TG, #T9033), tunicamycin (TM, #T7765), bafilomycin A1 (BafA1, #B1793), tumor necrosis factor-α (TNF-α, #T6674), torin1 (TOR, #475991), and staurosporine (STS, #S4400) were purchased from Sigma-Aldrich (St. Louis, MO, USA).

### High-content microscopy

Human osteosarcoma U2OS-GFP-LC3 wild type or TFEB and TFE3-double deficient (TF DKO), ATG5 deficient (ATG5^−/−^), RFP-GFP-LC3, RFP-LC3 wild type or mutant cells expressing a non-phosphorylatable knockin of eIF2α (eIF2α^S51A/S51A^) were seeded in 384-well µclear imaging plates (Greiner Bio-One) at a density of 2 × 10^3^ cells per well and allowed to adapt for overnight. Furthermore, ATG5^−/−^, eIF2α^S51A/S51A^, and TF DKO cells were treated for 6 h. Moreover, 2 × 10^3^ U2OS cells either wild type or stably expressing GFP-ATF6, CHOP::GFP, GFP-TFEB, or XBP1-ΔDBD-venus were seeded in 384-well black imaging plates (Greiner Bio-One) and let adhere overnight. Cells were then treated for 6 h to assess TFEB translocation, and 24 h to monitor abundance of ATF6 and spliced XBP1 (XBP1s), or to measure CHOP expression. Next, cells were fixed with 3.7% formaldehyde (#F8775; Sigma-Aldrich) supplemented with 1 μg/ml Hoechst 33342 (#H3570; Thermo Fisher Scientific) at 4 °C overnight. After washing the cells, the plates were sealed and analyzed by automated microscopy. Image acquisition was performed using an ImageXpress Micro XL automated microscope (Molecular Devices, Sunnyvale, CA, US) equipped with a ×20 PlanApo objective (Nikon, Tokyo, Japan), followed by automated image segmentation. A minimum of four images were acquired per well, and experiments involved at least triplicate assessments.

### Image segmentation and data analysis

Upon acquisition, images were segmented and analyzed using R. Briefly, cells were segmented and divided into nuclear and cytoplasmic regions based on the nuclear Hoechst staining and cytoplasmic GFP or RFP signal. After exclusion of cellular debris and dead cells, parameters of interest were normalized, statistically evaluated, and graphically depicted with R software. Using R, images were extracted and pixel intensities scaled to be visible (in the same extent for all images of a given experiment).

### Immunofluorescence

Cells were treated for 16 h to detect eIF2α phosphorylation (PeIF2α) and TFE3 expression, or 6 h to measure p65 nuclear translocation. Then cells were fixed with 3.7% PFA at 4 °C overnight. For the immunostaining of TFE3, p65, and phospho-eIF2α (Ser51), fixed cells were permeabilized with 0.1% Triton X-100 (#X100; Sigma-Aldrich) on ice, and unspecific antibody binding was blocked with 5% bovine serum albumin (BSA, w/v in PBS) for 1 h. Then cells were incubated with antibodies specific to TFE3 (#ab93808, 1:200; Abcam), phospho-eIF2 alpha (Ser51) (#ab32157, 1:1000; Abcam), or p65 (#4764, 1:100; Cell Signalling Technology) at 4 °C overnight. After washing with PBS twice, AlexaFluor 568-conjugated secondary antibodies (Thermo Fisher Scientific) were employed for additional 2 h at RT. Then cells were washed and imaged by automated fluorescence microscopy as described above. The nuclear-to-cytoplasm intensity ratio of TFE3 and p65 as well as the cytoplasmic intensity of phospho-eIF2α (Ser51) were measured and normalized to controls.

### Imaging cytofluorometric analysis

Six hundred microliters of total blood were diluted in 25 mL red blood cell lysis buffer (BioLegend) and incubated for 10 min at room temperature. Then the cells were washed twice in PBS, fixed with 4% PFA for 20 min at room temperature, permeabilized with 0.25% Tween-20 for 15 min at 4 °C, and blocked with 2% BSA in PBS. Cells were incubated with anti-phospho-eIF2 alpha (Ser51) and AlexaFluor 647-conjugated anti-mouse PTPRC/CD45 antibody (#clone 30-F11; BioLegend) for 1 h at room temperature. Then cells were incubated for 1 h with donkey AlexaFluor488-conjugated secondary antibody and Hoechst 33342 (0.5 µg/µL). Multispectral imaging flow cytometry was performed on an AMNIS ImageStream X Mark II equipped with 375-, 488-, 561-, and 642 nm lasers using the ×60 magnification lens. At least 6000 cells/sample were acquired for each sample. The analysis was performed with IDEAS software v6.2. Exclusively focused images were included in the analysis. Selection was based on the gradient RMS feature of bright field images. A compensation matrix was calculated using single color fluorescent controls. This matrix was applied to each file and singlets were then gated on aspect ratio vs. area of bright field and leukocyte subpopulations were gated on a pictogram indicating the intensity of PTPRC/CD45 staining vs. dark field. Following the intensity of peIF2α was quantified in each cell.

### Quantification of cell death by flow cytometry

Cell death was assessed by means of the Alexa Fluor 647 Annexin V (#640943; BioLegend) and DAPI (#62248; Thermo Fisher Scientific) kit following the manufacturer’s instructions. Briefly, cells were seeded in 12-well plates (with 5 × 10^4^ cells per well) and incubated at 37 °C in a humidified atmosphere with 5% CO_2_ for 24 h, then cells were collected and washed in PBS containing 0.5% BSA before the cell pellet was resuspended in 100 µL of Annexin V Binding Buffer (#422201, BioLegend) containing Alexa Fluor 647-coupled Annexin V. Samples were then incubated at room temperature in the dark for 15 min before adding 100 µL of PBS containing 0.5% BSA and 2 µg/mL DAPI solution. Acquisitions were performed on a BD LSRFortessa™ cell analyzer (BD Biosciences, San Jose, California, USA), and data were statistically analyzed using the FlowJo 10.5.3 software (Tree Star, Ashland, Oregon, USA).

### In vivo experimentation

The animal experiment was approved by the Gustave Roussy ethics committee with the project number: 24771–2020032413235413, and all procedures were performed in compliance with the governmental and institutional guidelines and regulations. Mice were kept in a temperature-controlled SPF environment (12 h light/dark cycles) with food and water ad libitum. Eight-week-old female C57Bl/6j mice were obtained from ENVIGO (France). To quantify the in vivo phosphorylation eIF2α (S51), naive mice were intraperitoneally (i.p.) treated with HCQ at a dose of 50 mg/kg/day in 200 µL PBS daily for 10 days^75,76^; fed with AZT in autoclaved drinking water at a concentration of 3 mg/L (purchased from the local pharmacy) for 5 days, and the solution was changed daily throughout the treatment period^77,78^. All mice were sacrificed at day 10, 4 h post-injection with HCQ, and livers and hearts were snap frozen in liquid nitrogen.

### Immunoblotting

Thirty milligrams of liver tissue were dissociated in Precellys lysing tubes (#CK28; Bertin Technologies SAS, France) containing 1 mL of RIPA lysis buffer (#89901; Invitrogen) by using the Precellys 24 homogenizer (Bertin Technologies SAS) at 6500 r.p.m. for 60 s, followed by spinning at 14 s 10^3^ d *g* for 15 min to collect the supernatant that contains soluble proteins. Protein concentration was measured by means of by the BCA Assay (Bio-Rad, Hercules, CA, USA). The protein solution was mixed with 4× loading buffer (#NP0008; Invitrogen), and denatured at 100 °C for 10 min before subjected to western blotting. Forty micrograms of total protein were resolved on 4–12% NuPAGE Bis-Tris protein gels (#NP0336BOX; Invitrogen) and transferred to PVDF membranes (#IPFL00010; Merck Millipore). The membranes were blocked with 5% non-fat dry milk in TBST for 1 h before incubating with primary antibodies to phospho-eIF2 alpha (Ser51) (#ab32157, 1:1000; Abcam) overnight at 4 °C. Membranes were washed several times with TBST for 10 min each before incubation with HRP-conjugated secondary antibody (#4050-05; SouthernBiotech) for 2 h at room temperature. At last, the membranes were washed again and subjected to chemiluminescence detection with the Amersham ECL Prime detection reagent kit (#RPN2236; GE Healthcare) on an ImageQuant LAS 4000 software-assisted imager. The exposed membranes were stripped and re-probed with antibodies specific to β-actin (#ab20727; Abcam) as loading control using the procedure described above. Densitometry was performed using the ImageQuant TL software (GE Healthcare, Piscataway, NJ, USA).

### Image and data processing

Images were segmented using the EBImage package (available from Bioconductor repository https://www.bioconductor.org) with the R software. The nuclear region was defined using a polygon mask based on the nuclear Hoechst signal, and a second polygon mask was generated using the cytoplasmic GFP or RFP signal. For the assessment of autophagic vesicles, a third mask was created on cytoplasmic regions exhibiting a high intensity signal of GFP or RFP corresponding with LC3 aggregates.

Following image segmentation, the data were extracted and reduced using the R software. After exclusion of cellular debris and dead cells, parameters of interest were normalized to controls, statistically evaluated, and graphically depicted with R software. Using R, images were extracted and pixel intensities scaled to be visible (to the same extent for all images of a given experiment).

### Statistical analysis

Unless otherwise mentioned, data are reported as means ± SD of triplicate determinations and experiments were repeated at least three times yielding similar results, and statistical significance was assessed by Student’s *t*-test with a *P* value adjustment based on the Benjamini–Hochberg procedure.

## Supplementary information

Supplemental Figure Legends

Supplemental Figure 1

Supplemental Figure 2

Supplemental Figure 3

Supplemental Figure 4

Supplemental Figure 5

Supplemental Figure 6

Supplemental Figure 7
