# Supplementary material for: Lysosomotropic agents including azithromycin, chloroquine and hydroxychloroquine activate the integrated stress response
Source: Cell Death Dis. 2021 Jan 6;12(1):6. doi: 10.1038/s41419-020-03324-w (PMC7790317; doi:10.1038/s41419-020-03324-w)
Supplement: Supplementary file 1 — Supplemental Figure Legends [file 41419_2020_3324_MOESM1_ESM.docx]

## Supplemental figures

**Figure S1. Chloroquine, hydroxychloroquine and azithromycin induce the formation of LC3 puncta in human glioma H4. (A,B)**. Human glioma H4-GFP-LC3 wild-type or ATG5 knockout (KO) were treated with chloroquine (CQ; 10, 20, 40 μM), hydroxychloroquine (HCQ; 10, 20, 40 μM) or azithromycin (AZT; 10, 20, 40 μM) for 6 h. Torin 1 (TOR) at 300 nM was used as positive control for autophagy induction and bafilomycin A1 (BafA1) at 100 nM was used as a prototype inhibitor of autophagic flux. After fixation, GFP-LC3 dots were analyzed as a proxy for autophagy induction. Representative microscopical images are shown in **A** (AZT, CQ and HCQ, 40 µM) and normalized mean data are depicted as a bar chart in **B**. Scale bar equals 10 μm. Data are means ± SD of 4 replicates (****P* < 0.001 vs. vehicle control (Ctrl) and ^###^*P* < 0.001 *vs*. WT; Tukey’s multiple comparisons test).

**Figure S2. Chloroquine, hydroxychloroquine and azithromycin inhibit autophagic flux. (A,B)**. Human osteosarcoma U2OS RFP-GFP-LC3 cells were treated with chloroquine (CQ; 10, 20, 40 μM), hydroxychloroquine (HCQ; 10, 20, 40 μM) or azithromycin (AZT; 10, 20, 40 μM) for 6 h. Torin 1 (TOR) at 300 nM was used as positive control for autophagy induction and bafilomycin A1 (BafA1) at 100 nM was used as a prototype inhibitor of autophagic flux. After fixation, RFP^+^GFP^+^ and RFP^+^GFP^-^ LC3 dots were analyzed and the autophagy inhibition score was calculated. Representative microscopical images are shown in **A** and normalized mean data are depicted as bar chart in **B**. Scale bar equals 10 μm. Data are means ± SD of 4 replicates ((**p*<0.05, ***p*<0.01,****P* < 0.001 vs. vehicle control (Ctrl) Student’s *t*-test).

**Figure S3. Chloroquine, hydroxychloroquine and azithromycin induce the phosphorylation of eIF2α. (A,B)**. Human osteosarcoma U2OS cells were treated with chloroquine (CQ; 40 μM), hydroxychloroquine (HCQ; 40 μM) or azithromycin (AZT; 40 μM) for 6 h. Thapsigargin (TG, 3 μM) was used as a positive control for ER stress induction. Cells were collected, lysed and proteins were separated by chromatography. After immobilisation on membranes proteins were detected by antibodies specific to phosphorylated eIF2α (peIF2α), eIF2α or β-actin (ACTB). Representative immunoblot images are shown in **A** and the normalized ratios of peIF2α/eIF2α are depicted as bar chart in **B**. Data are means ± SD of replicate experiments (**p*<0.05, ***p*<0.01 vs. vehicle control, Ctrl, Student’s *t*-test).

**Figure S4. Chloroquine and hydroxychloroquine induced the nuclear translocation of p65.** A-B. U2OS cells were treated with chloroquine (CQ; 10, 20, 40 μM), hydroxychloroquine (HCQ; 10, 20, 40 μM) and azithromycin (AZT; 10, 20, 40 μM) for 6 h, then the cells were fixed with 3.7 % paraformaldehyde (PFA, w/v in PBS). Tumor necrosis factor alpha (TNFα; 40 nM) was employed as positive control. The translocation of p65 was assessed by means of immunofluorescence staining using a specific antibody and representative images are depicted in **A**. p65 nuclear and cytoplasmic intensity were measured, and the ratio of p65 intensities in nucleus and cytoplasm was calculated to indicate p65 nuclear translocation (**B**). Scale bar equals 10 μm. Data are means ± SD of 4 replicates (**P* < 0.05, ****P* < 0.001 vs. DMSO/Ctrl, Student’s t-test).

**Figure S5. eIF2α phosphatase inhibitors increase the phosphorylation of eIF2α. (A,B)**. Human osteosarcoma U2OS cells were treated with 5 mM 4-phenylbutyric acid (4-PBA), 10 µM nelfinavir (NFV), 25 µM salubrinal (SAL) or were left untreated for 6 h. Thapsigargin (TG, 3 μM) was used as a positive control for ER stress induction. Cells were collected, lysed and proteins were separated by chromatography. After immobilisation on membranes proteins were detected by antibodies specific to phosphorylated eIF2α (peIF2α), eIF2α or β-actin (ACTB). a Representative immunoblot images are shown.

**Figure S6. Chloroquine- and hydroxychloroquine-induced cell death.** Human osteosarcoma U2OS WT or eIF2α^S51A^ knock in were exposed to 10, 20, 40 μM chloroquine (CQ) or hydroxychloroquine (HCQ). Annexin V/DAPI staining was used to assess cell death by flow cytometric analysis and representative dot plots are shown. Staurosporine (STS) at 2 μM was used as positive control for apoptotic cell death induction.

**Figure S7. Effects of hydroxychloroquine plus azithromycin in vivo.** Mice were treated *intraperitoneally* (*i.p.*) with 50 mg/kg/day hydroxychloroquine (HCQ), orally supplemented with azithromycin (AZT) (3 mg/L drinking water) or their combination as illustrated in the scheme (**A**). Hearts were excised from 3 mice of each group at the end of treatment and subjected to protein extraction for SDS–PAGE and immunoblot to detect the phosphorylation of elF2α (**B**). β-Actin (ACTB) was used as a loading control. Band intensities were quantified and the ratio of pEIF2a to ACTB was calculated. Data are expressed as means ± SEM of three mice (**C**).
