# Supplementary figures and images for: Lysosomotropic agents including azithromycin, chloroquine and hydroxychloroquine activate the integrated stress response
Source: Cell Death Dis. 2021 Jan 6;12(1):6. doi: 10.1038/s41419-020-03324-w (PMC7790317; doi:10.1038/s41419-020-03324-w)

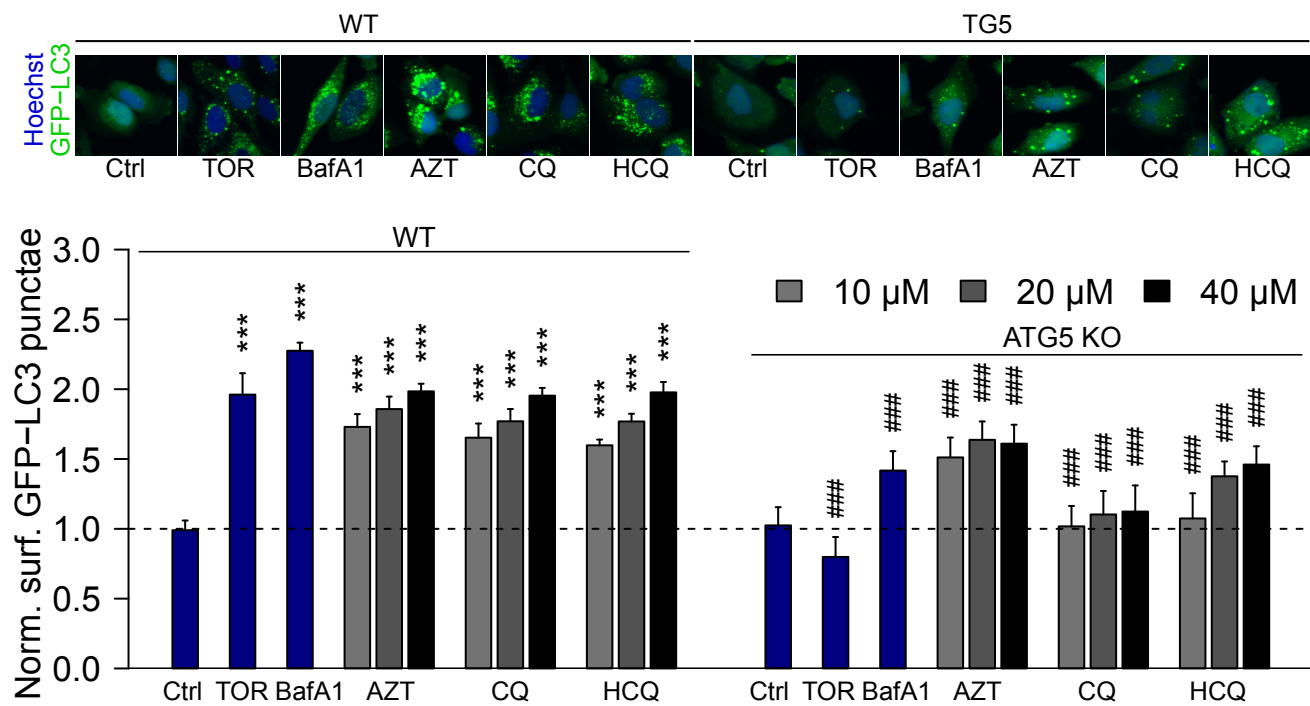

**Fig. S1**

Supplement: Supplementary file 2 — Supplemental Figure 1 [file 41419_2020_3324_MOESM2_ESM.pdf]

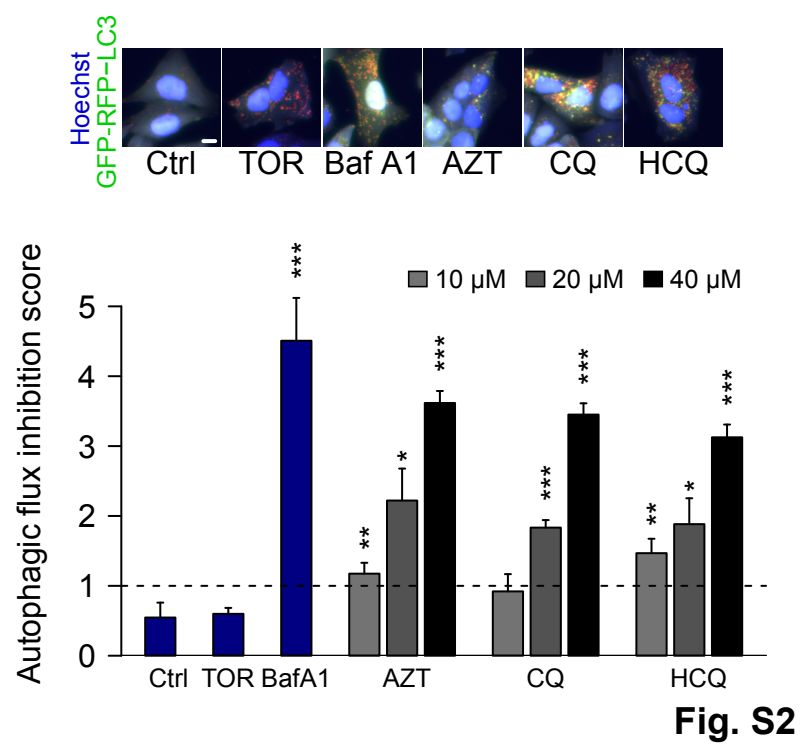

Supplement: Supplementary file 3 — Supplemental Figure 2 [file 41419_2020_3324_MOESM3_ESM.pdf]

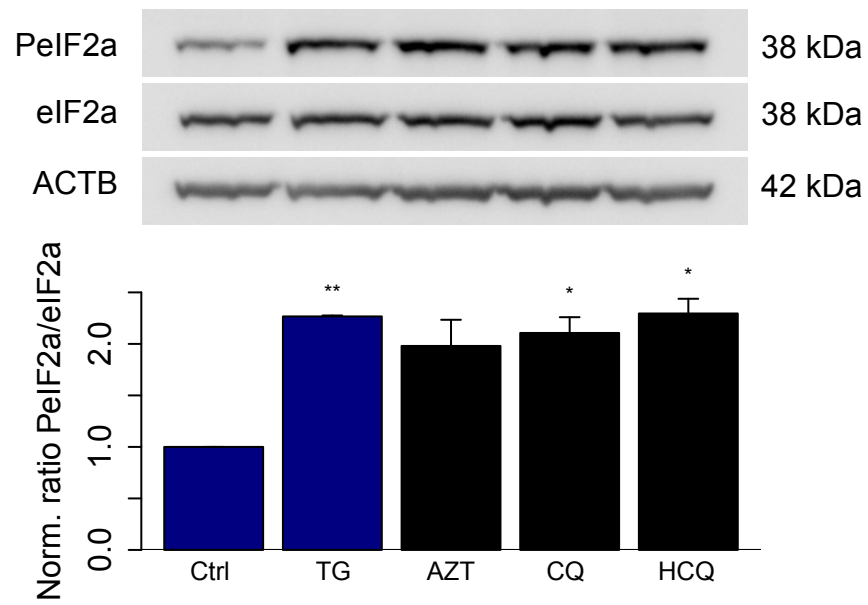

**Fig. S3**

Supplement: Supplementary file 4 — Supplemental Figure 3 [file 41419_2020_3324_MOESM4_ESM.pdf]

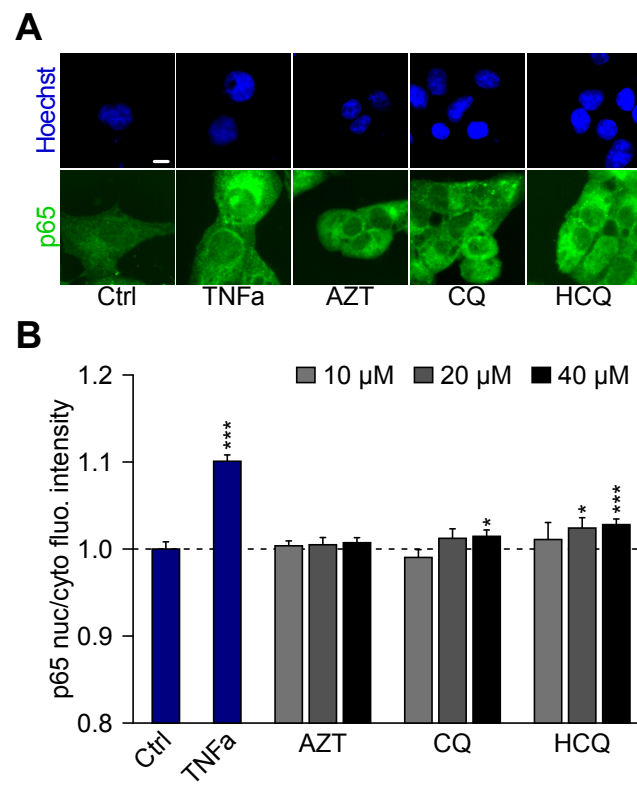

**Fig. S4**

Supplement: Supplementary file 5 — Supplemental Figure 4 [file 41419_2020_3324_MOESM5_ESM.pdf]

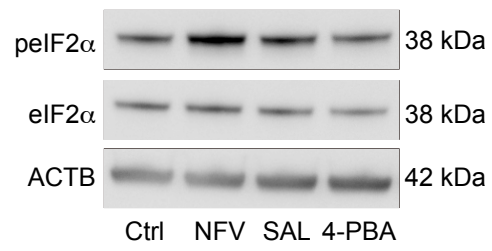

**Fig. S5**

Supplement: Supplementary file 6 — Supplemental Figure 5 [file 41419_2020_3324_MOESM6_ESM.pdf]

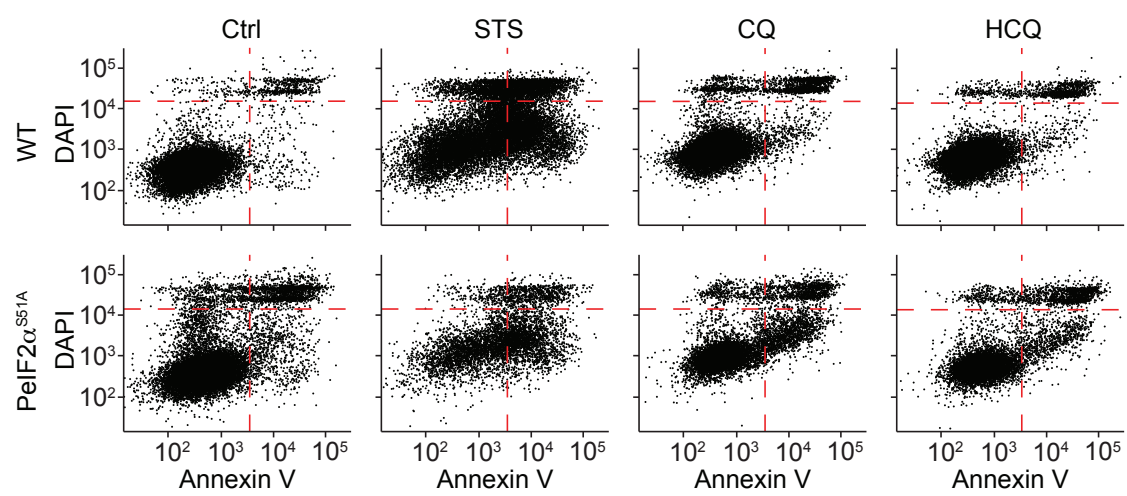

**Fig. S6**

Supplement: Supplementary file 7 — Supplemental Figure 6 [file 41419_2020_3324_MOESM7_ESM.pdf]
