# Supplementary material for: Lysosomotropic agents including azithromycin, chloroquine and hydroxychloroquine activate the integrated stress response
Source: Cell Death Dis. 2021 Jan 6;12(1):6. doi: 10.1038/s41419-020-03324-w (PMC7790317; doi:10.1038/s41419-020-03324-w)
Supplement: Supplementary file 8 — Supplemental Figure 7 [file 41419_2020_3324_MOESM8_ESM.pdf]

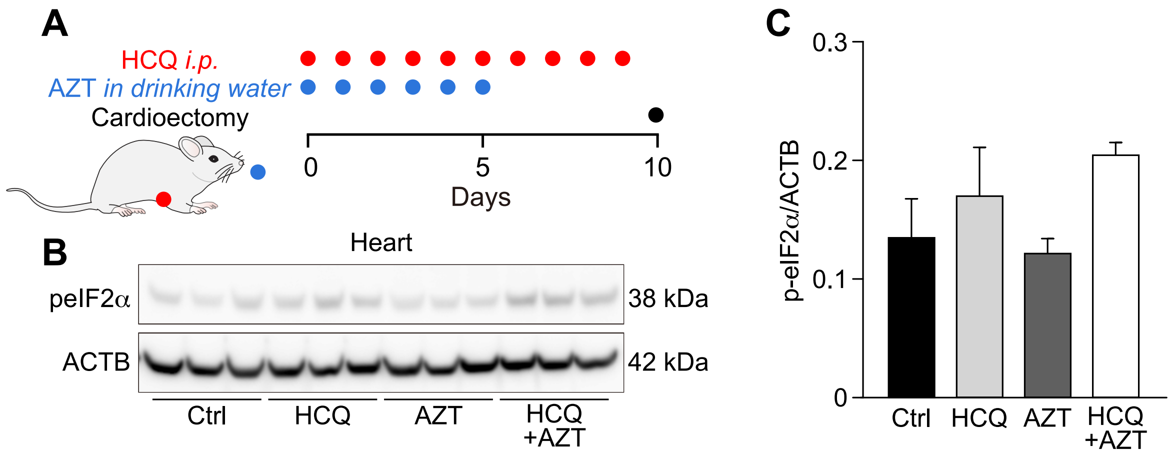
A
HCQ i.p.
AZT in drinking water
Cardioectomy
0
5
10
Days
B
Heart
peIF2α
ACTB
38 kDa
42 kDa
Ctrl
HCQ
AZT
HCQ +AZT
C
p-eIF2α/ACTB
0.3
0.2
0.1
0
Ctrl
HCQ
AZT
HCQ +AZT

Fig. S7
